# Educational outcomes of a new curriculum on interproximal oral prophylaxis for dental students

**DOI:** 10.1371/journal.pone.0204564

**Published:** 2018-10-10

**Authors:** Denis Bourgeois, Ina Saliasi, Claude Dussart, Juan Carlos Llodra, Delphine Tardivo, Laurent Laforest, Manuel Bravo, Stéphane Viennot, Bruno Foti, Florence Carrouel

**Affiliations:** 1 Laboratory Systemic Health Care, EA4129, University Lyon, Lyon, France; 2 Faculty of Dental Medicine, University Lyon, Lyon, France; 3 Faculty of Dental Medicine, University of Granada, Granada, Spain; 4 Laboratory Anthropology, Health Law, and Medical Ethics, UMR 7268, University Aix Marseille 2, Marseille, France; University of Queensland, AUSTRALIA

## Abstract

**Objective:**

The aim of this study was to evaluate the effectiveness of a preclinical oral prophylaxis education program by examining the effectiveness of the teaching module on changes to the students’ attitudes towards their individual hygiene behaviors with interdental brushes (IDBs).

**Methods:**

As being part of a new didactic program on oral interproximal prophylaxis, all preclinical third-year students (n = 96) enrolled in the 2014/15 academic year received theoretical, preclinical, and clinical lessons on interproximal prophylaxis. The evaluation of educational outcomes was linked to observed changes in students' hygiene behaviors using interdental brushes. Knowledge, skills, attitudes, satisfaction, competence and performance were also explored. The evaluation interviews were recorded at each recall, i.e., 1 week, 1 month, 3 months and 1 year after baseline.

**Results:**

Motivation to use IDBs is clearly related to the perception of the effectiveness of the brushes and the perception of bleeding reduction. At one week, 89.6% of subjects reported using IDBs. Individual use decreased significantly from one week to one month (-26%, p *=* 0.006) while a non-significant upward trend occurred between one month and three months. Among students reporting usage of IDBs at 1 year (20.8%), only 2.0% used IDBs daily. Most students would recommend IDBs to other people at the beginning (69.8%). However, this share dropped to 50% at 3 months. IDB-users prescribed more than non-users.

**Discussion and public health implications:**

The implementation of a module on interdental hygiene practices in the oral health program is strongly recommended. However, corrective measures should be considered regarding the organization and frequency of recall periods in order to improve the performance of the curriculum.

## Introduction

Oral conditions, as chronic diseases, are highly prevalent, affecting 3.9 billion people. Severe periodontitis conditions evaluated for the entire Global Burden of Diseases 2010 Study were the 6th most prevalent conditions, affecting 11% of the global population [1]. Periodontal disease is a component of the global burden of chronic disease [2]. It is characterized at its onset by a synergistic and dysbiotic microbiota in which different members or specific gene combinations fulfill distinct roles that converge to shape and stabilize a disease-provoking microbiota [3].

In oral health, the interdental spaces are an ecological niche for which the body has few or no defenses and for which the traditional daily methods for control via disrupting biofilm as twice daily tooth brushing are inadequate. In young healthy adults, the interdental space holds approximately 16 billion bacteria, including *P*. *gingivalis*, a pathogen for heart disease and other systemic diseases [4,5]. Evidence suggests that the oral biofilm can have important health outcomes, such as periodontitis, and potential associations with and/or causes of cardiovascular disease, diabetes, chronic kidney disease, degenerative disease, and depression [5,6].

Originally, interdental brushes (IDB) were recommended by dental professionals to patients with large embrasure spaces between the teeth [7], caused by the loss of interdental papilla mainly due to periodontal destruction. Patients who had interdental papillae that filled the embrasure space were usually recommended to use dental floss as an interdental cleansing tool. Flossing cannot be recommended other than for sites of gingival and periodontal health, where IDBs will not pass through the interproximal area without trauma. Otherwise, IDBs are the device of choice for interproximal plaque removal [8]. The recent workshop consensus by the European Federation of Periodontology concluded “flossing cannot be recommended other than for sites of gingiva and periodontal health, where interdental brushes will not pass through the interproximal area without trauma”. “In young individuals in whom the papillae fill out the interdental spaces, dental floss is the only tool which can reach into this area” [9]. One challenge, our challenge is to go against this generally induced idea that IDBs do not fall within the scope of individual prophylaxis in healthy adults whose accessibility is made difficult [4]. Consequently, they are mainly considered when the clinical signs of periodontal lesions appear in adult patients [8]. It is also important that further well designed and appropriately powered clinical trials are warranted to provide more guidelines on interdental oral health selection [10].

In daily oral hygiene activities, it is very difficult to reach this space and disrupt the biofilm of a healthy adult especially in the posterior parts of the mouth [10]. However, updated recommendations for tools and techniques have been recently identified for interdental cleaning, and these approaches aim to reduce interproximal plaque, reduce bleeding, control gingivitis and increase patient motivation and compliance for a more effective prophylaxis and a better quality of life [11]. Accessibility of interdental spaces of healthy adults with the use of calibrated IDBs has been demonstrated [5]. Conventional tooth brushing alone is not effective in removing the biofilm within interdental spaces [8,10]. Thus, interdental cleaning, similarly to toothbrushing, should become an established part of daily oral hygiene.

In the past decades, oral prophylaxis training in dental faculties has mainly focused on brushing alone or the combined use of brushing and dental floss [12]. Similarly, there have been no major actions to increase levels of knowledge or skills in interdental cleansing in the general population. Global oral health programs rarely include this topic [13].

As a key stakeholder in the oral health of their future patients and recognizing the importance of good interdental prophylaxis, a specific curriculum was developed for dental students within the Public health program of the faculty of Lyon. Introduced from the third year of preclinical study, it aims to focus on the major role of IDBs, as well as dental brushing and toothpastes, in the dental education of students. Therefore, the Individual Interproximal Oral Prophylaxis Education Program (IIOPEP) was developed at the University of Lyon to highlight the need for interdental prophylaxis education and to evaluate the effectiveness of this program targeted at a cohort of “dentists of tomorrow”.

The aim of this follow-up study was to evaluate the effectiveness of a preclinical oral prophylaxis education program with respect to observed changes in the individual hygiene behaviors of students regarding the use of IDBs. Interdental bleeding in students, along with the correlates of their IDB perceptions, recommendations to relatives and prescriptions to patients were also explored at each recall, i.e., 1 week, 1 month, 3 months and 1 year after baseline.

## Materials and methods

### Ethical approval

Classified as non-interventional research and a teaching practices investigation, the study does not, under national law, require ethics committee approval or written informed consent (www.legifrance.gouv.fr/eli/decret/2016/11/16/AFSP1621392D/jo/texte). Regional ethics committee approved the protocol (Rech_FRCH_2013/0023). The French data protection authority (CNIL) indicated that we were not required to declare the survey to the French authority that oversees the protection of personal data (S1 Material).

### Study population

All third-year (n = 96) pre-clinical students enrolled in the 2014/15 academic year in the Faculty of Oral Medicine of Lyon followed didactic, preclinical (hands-on experience using manikins), and clinical (hands-on experience on patients) learning throughout the curriculum. In France, dentistry courses are organized into 12 semesters taken over six years. The entire curriculum is organized on the principle of flipped pedagogy, a pedagogical model in which the typical lecture and homework elements of a course are reversed. Lectures are viewed by students at home before the class session, while in-class time is devoted to exercises, projects, or discussions.

Students have already performed one year of medical studies common to the studies of dentistry, medicine, pharmacy and midwifery. At the end of their third year, they start clinical work with patients in hospitals, which involves semiology, prevention consultation, periodontics, surgery, dentistry and dentures. In each of these disciplines, they can thus apply the lessons learned in individual oral prophylaxis. All anonymous data was collected as part of the students' compulsory curriculum. This is part of the traditional process of evaluating teaching within the faculty. No additional data was recorded for the purposes of the study. With respect to the research on the use of a new curriculum on interproximal oral prophylaxis, the students were not aware of the intention to communicate the evaluation of the results.

### Content and development of the learning module

**Fig 1:**
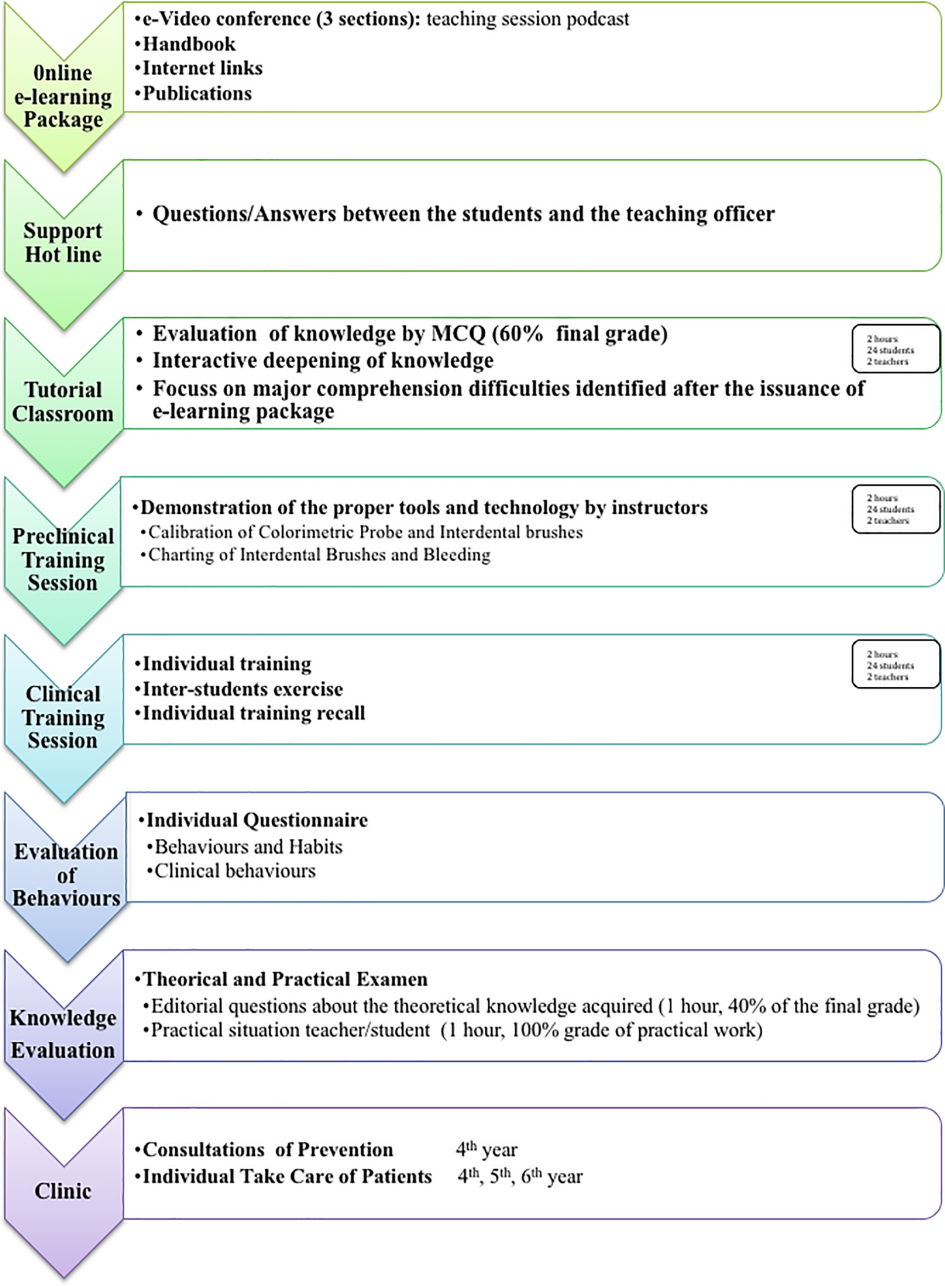
Workflow of the evaluation.

The workflow of the module, including the five-stage technique “*Online e-learning tutorial*, *Organization of a tutorial classroom*, *Advanced preclinical training session on interdental oral prophylaxis practices*, *Clinical training session and Recall clinical training sessions”* of skill acquisition, is described in Fig 1. The details of the curriculum are presented in Table 1. Briefly, the 3 first steps provided students with comprehensive knowledge and understanding of the issues related to interdental biofilm and how to address them. The fourth step consisted of a practical clinical training session to promote efficient scoring and management of biofilm health consequences in dental clinical practice. Students were also sensitized regarding the content and the way to deliver the prevention messages to their patients. The last phase was conducted with recall sessions until the 12^th^ month following the implementation of the curriculum (Table 1). The module including all recalls was mandatory as it is implemented in the curriculum of the faculty. It was explained orally and individually to the students that nothing obliged them from baseline to implement for themselves the clinical lessons received. The students had total freedom to implement or not the process of using IDBs without prejudice or value judgment against them.

**Table 1:** Description of the successive steps of the curriculum.

**preliminary didactic and preclinical phases (1–3)**		
Steps	Learning objectives	Contents
***Provision of the online e-learning tutorial (1)*** E-learning package sent to students via email	• To describe the current concept of prophylaxis given the complexity of the interdental biofilm and its effect on systemic health • To propose criteria for the proper choice of interdental techniques	• E-video conference sequenced into 3 20-minute chapters: a. Individual prophylaxis and biofilm disorganization; b. Biofilm: Tools and Methods of disorganization; c. Biofilm: Interdental spaces, Bacteria and Bleeding • A handbook to assess the interdental spaces with case studies, open questions and appropriate iconography • Internet links to analyze advertisements and/or interdental techniques • Recommendations of publications supporting the scientific rationale of the prophylactic concept • E-Learning module: Over 15 days, each student could ask the teaching supervisor via an online forum about issues or topics
***Organization of the tutorial classroom (2)*** Directed teaching of 2 hours in groups of 24 students	• To describe and demonstrate basic concepts of biofilm technology • To identify technical innovations that meet the criteria for technical feasibility, cost, and individual desirability/usability of an interdental prophylaxis technology • To formulate qualitative questions that connect the fundamentals to clinical innovation objectives	• Prior learning assessment by multiple-choice questions (MCQs) with a voting box • Interactive deepening of knowledge related to the learning objectives • Session restricted to major comprehension difficulties identified after the issuance of the e-learning package
***Advanced preclinical training session on interdental oral prophylaxis practices (3)*** IAP colorimetric probe with a color code provided to students Sessions organized by 2 highly experienced instructors in groups of 24 students	• To explain the rationale for evaluating the access diameter of the interdental spaces. • To demonstrate how to use the right interdental brush in clinical practice • To estimate how to record interdental bleeding in clinical practice	• Students received a package including: . an IAP colorimetric probe to evaluate an access diameter defined by the gauge of the wire core CURAL as 5 cylindrical CPS IDBs (Curaprox CPS, Curaden International AG, Kriens, Switzerland) (S1 Fig) . a color code related to the size of the brush and screening cards • Demonstration to the students by instructors about proper tools and technology
**practical clinical phase (4) and maintenance phase (5)**		
Steps	Learning objectives	Contents
***Clinical training session (4)*** . Interproximal Clinical Examination . Training clinical session Session organized by 2 highly experienced instructors in groups of 24 students	• Organize clinical sequences for students to take control of their oral health to disrupt the biofilm of the interdental spaces with personal acquisition of the prophylaxis technique • Assess their bleeding gingival score	• The Interproximal Clinical Examination, a scoring method including the classification of the access diameter for interdental space, and the internal validity of the examiners to record bleeding conditions were described in detail in *«Access to Interdental Brushing in Periodontal Healthy Young Adults: A Cross-Sectional Study » (4)* • Individual training session with the correction of the interdental prophylaxis technique, if necessary • Inter-students exercise with rotation (Patient/Student/Assistant Secretary) • Recommendations on how to implement the “Touch to teach interdental students’ training”, in terms of the method of education and communication, were individually installed. In the Touch-to-Teach method, the student partner holds his colleague's interdental toothbrush. Through hands-on training on the use of selected tools and techniques, he can better know, have a better idea of where and how the IDB cleans. The student-patient is guided and accompanied in his gesture by the instructor. He also learns to recalibrate several times in his technique so as not to use excessive pressure. It covers a non-traumatic way of learning good oral care. Training follows this instruction on the clinical use of the colorimetric probe to assess the diameter of each interdental site. The record charting includes for each site the interdental diameter and bleeding score
***Recall clinical training sessions (5)*** at: • 1 week (T1) • 1 month (T2) • 3 months (T3) • 1 year (T4)	• To assess the effect of a preventive recall strategy on changes in behaviors and attitude level over time at post- and recall visits • To reinforce self-care instructions and compliance	• Sessions included repetition of practical hand, re-motivation, error management, re-calibration, and evaluation of clinical performance based on the evolution of the bleeding score • At the end of the sessions, students had a new IDB kit available with the opportunity to receive free additional brushes in case they were needed between each individual recall

### Evaluation of the intervention: Outcomes

The different outcomes were collected at 1 week (7 days, T1), 1 month (4 weeks, T2), 3 months (12 weeks, T3) and 1 year (48 to 52 weeks, T4).

#### Primary outcome

Our main outcome was the percentage of IBD-users immediately before each time-point. IDB-use is a central variable in this study, and conceptually to use the IDB is the expected output of this study. Nevertheless, it is not a limitation, in our opinion, to consider in some statistical analysis this variable as independent. For example, in our analysis it plays mainly a role of an independent variable or factor predicting other variables. The declared frequency in use was also detailed into 6 mutually exclusive categories: no use, < 1 per week, 1/3 days, 1/2 days, daily, and more than daily. The reasons for non-use (lack of motivation, information on how to use, accessibility problems, pain, discomfort and cost) were rated by specific Likert scales scored from 1 (total agreement) to 5 (total disagreement). Respondents also have the option of saying, "Do not know". When calculating, the "Do not know" responses were treated as missing values. Data from Likert scales were treated as interval data.

#### Secondary outcomes

Secondary outcomes were perceptions of IDB use in terms of acceptability, traumatism (pain, iatrogenia, sensibility) and efficacy, rated with Likert scales scored from 1 (very good) to 5 (very bad). This 3 criteria have priority to ensure the patient's engagement [14–16]. The latter requires the practice of the good hygiene methods taught on the disorganization of the interdental biofilm and use of the material recommended by the practitioner. These were assessed in IDB users only. Student recommendations of IBD use to their community (T1 to T4) and prescription to their patients (T4) were also appraised. Lastly, students reported their perception of their interdental bleeding, perceived bleeding reduction (Likert scale scored from 1: “Often” to 4: “Never”) and health outcome, which was the percentage of bleeding interdental sites for each student.

### Collection of data

At the beginning of each session (T1, T2, T3, T4), a questionnaire (S2 Material) was given to students. Afterwards, students underwent oral examinations by 2 examiners to determine the percentages of bleeding interdental sites. The Bleeding on Interdental Brushing Index was recorded, as was the bleeding response to the horizontal pressure applied in the interdental area by a calibrated IDB [17]. After 30 s, bleeding at each gingival unit was recorded according to the following scale: 0, absence of bleeding after 30 s; and 1, bleeding after 30 s [18]. A gold standard examiner (DB), specialist in epidemiology, and with vast experience in screening, led calibration stages including the record of baseline characteristics. The 2 examiners obtained a minimum kappa value of 0.84 compared to the gold standard examiner. The questionnaire was also evaluated through a convenience sample from dental students (N = 28). Interexaminer concordance was also good, as the kappa coefficient was higher than 0.76 for the majority of items (range: 0.68 to 0.95).

### Statistical analysis

After a preliminary description of the characteristics of the students, the changes were assessed for each outcome between the different time-points following the curriculum from T1 to T4, using both global and pair-wise comparisons. As no student used IDB before the curriculum, the baseline period was not considered. Then, linear regression models were conducted for each quantitative outcome to determine the potential influence of factors measured at baseline before implementing the curriculum IDB use at the preceding time-point, gender, tobacco use and percentage of bleeding sites at baseline. All factors were forced in the different models. Linear regression models were conducted for each quantitative outcome variable (and at each time-period) to determine the association with some baseline factors: sex, tobacco and bleeding, together with IDB-use in that time-period (for example, for acceptability at 1-month, IDB-use were tested in the time-period between 1-week and 1-month, etc). Those factors were forced, for being potentially confounding factors. We did not statistically test the assumptions (linearity, homocedascity and normality), of those regression models, for the high risk of multiple testing (many regression models) and for this technique (linear regression), as it is well-known, being very robust. Instead, we decided “a visu”, looking at the residuals distributions graphs looking for outlayers or high deviations that could affect this robust technique. No problem was found for applying the models. Furthermore, as a further checking, we did not find in the distribution of the studied variables any atypical value separated more than 3 times the interquartile range, from Q1 (first quartile) or Q3 (third quartile).

SPSS Windows 20.0 (IBM, Chicago, IL, USA) was used for the descriptive statistics (mean values with SD and percentages) and analytical statistics (p-values calculation) in those analyses with the student as the unit of analysis. Models (binary logistic regression) for dichotomous variables were used. SUDAAN 7.0 (Research Triangle Institute, Research Triangle Park, NC) was used for analytical purposes (P value calculation) to make use of all available data in those repeated measurements situations (see Table's footnotes). SUDAAN enables to obtain, after the application of the Taylor series method [19], consistent estimates of standard errors for cluster-correlated or repeated measurements data. The detailed statistical methods are indicated in the table footnotes. All data were considered statistically significant when p < 0.05.

## Results

### Baseline characteristics

All 96 third-year students completed the survey. Of these students, 43.8% were female and 56.3% were male; 24% used tobacco. Their average age was 21.6 ± 2.1 years. All reported brushing their teeth at least twice per day. No subject reported experience with the use of dental floss and/or the use of interdental brushes.

The mean number of teeth was 29.0 ± 1.3. Missing teeth were due to orthodontic extractions (2.8%) and absence of the third molars (97.2%). As far as clinically visible, all participants were free of active caries and interproximal caries.

In the study, 40.5 ± 25.1% of the interdental sites had bleeding and 100% of students had access to IDBs. IDBs can penetrate in 94.5% of interdental spaces with 80.4% indicating brushes with a small diameter (0.6–0.7 mm).

### Changes in interdental hygiene behavior

One week after baseline, 89.6% of subjects reported using interdental brushes. Individual use decreased significantly from 1 week to 1 month (percentage change –26.1, p *=* 0.006), while a non-significant upward-trend appeared from 1 month to 3 months following baseline (percent change +9.40, p = 0.06). In contrast, IBD use markedly decreased at one year. The effect for the 1-year (48 to 52 weeks) time point differs significantly and converts back to a 52.1% reduction. A total of 50 out of 70 students who reported using IDB at 3 months reported stopping at the twelfth month (Table 2).

**Table 2:** Change over time of indicators in dental students (n = 96).

Variable	1 week [T1]		1 month [T2]		3 months [T3]		1 year [T4]		Global p-value	Paired Comparisons^i^
	n	value	n	value	n	value	n	value		
Use (% of all categories) of IDB (last period)^a^	96	10-6-6-29-20-25-3	96	36-6-13-25-12-7-0	96	27-5-28-20-11-8-0	96	79-3-9-4-2-2-0	<0.001^e^	T1≠T2T3≠T4^j^
**Detail of Non-Users/Users (last period), n (%)**									<0.001^f^	T1≠ T2 T3≠ T4^k^
No	10	(10.4)	35	(36.5)	26	(27.1)	76	(79.2)		
Yes	86	(89.6)	61	(63.5)	70	(72.9)	20	(20.8)		
**Reason for not using IDB, mean±sd [IDB-non-users]**											
(Likert scale 1 = total agree to 5 = total disagree)										
Motivation (Determination)	10	1.80±0.92	26	2.27±1.25	24	1.96±1.08	67	3.61±1.03	<0.001^g^	*
Ability to use IDB	10	3.80±0.63	24	3.96±0.91	19	4.11±0.57	68	3.13±1.17	<0.001^g^	*
Accesibility problem	9	2.78±1.39	24	3.08±1.25	19	3.00±0.94	66	2.82±1.16	0.583^g^	*
Pain	10	2.30±1.34	25	2.68±1.49	21	2.14±0.91	67	2.87±1.27	0.018^g^	*
Bleeding	10	2.70±1.49	25	2.76±1.48	21	2.10±0.89	67	3.09±1.22	<0.001^g^	*
Too uncomfortable	10	2.20±1.14	25	2.28±1.31	22	2.00±0.93	67	2.16±1.07	0.715^g^	*
Cost		-		-		-	62	3.05±1.30	-	
**Evaluation of IDB, mean±sd [IDB-users]**										
(Likert scale 1 = very good to 5 = very bad)										
Acceptability	86	2.27±0.87	61	2.10±0.75	70	2.19±0.79	20	2.15±0.75	0.153^g^	-
Traumatism (sensibility, pain, iatrogenia)	86	2.85±1.00	61	2.62±0.90	70	2.63±0.84	20	2.40±0.75	0.013^g^	T1≠T2^l^
Perceived efficacy	86	1.63±0.67	60	1.53±0.65	70	1.97±1.02	19	1.79±0.63	0.006^g^	T1T2≠T3^l^
**Have you recommended IDB use?, valid total n (% Yes)**										
All	96	(69.8)	96	(69.8)	96	(50.0)	96	(90.6)	<0.001^f^	T1T2≠T3≠T4^k^
IDB-non-users	10	(40.0)	35	(57.1)	26	(46.2)	76	(88.2)	<0.001^h^	T2T3≠T4^n^
IDB-users	86	(73.3)	61	(77.0)	70	(51.4)	20	(100)	<0.001^h^	T1T2≠T3≠T4^n^
p-value^b^		0.062		0.070		0.818		0.197		
**How many people**^c^ **have you recommended IDB use?, mean±sd**										
All	96	2.83±2.98	96	2.83±2.98	96	1.60±2.23	96	11.50±15.30	<0.001^e^	T1T2≠T3≠T4^m^
IDB-non-users	10	1.70±3.16	35	1.99±2.57	26	1.50±2.34	76	11.18±16.25	<0.001^g^	T1T2T3≠T4^l^
IDB-users	86	2.96±2.95	61	3.31±3.10	70	1.64±2.21	20	12.80±11.30	<0.001^g^	T1T2≠T3≠T4^l^
p-value^d^		0.204		0.033		0.779		0.680		

a: It includes percentages with no decimals, for categories: No;Less-1/week;1/3 days;1/2 days;1/day;More. I.e., at T1 (1 week), the percentages were, respectively: 10% (no brush), 6% (less frequency than 1/week), etc. Last period refers to the immediately previous period, i.e., between 1 week and 1 month for T2

b: Chi square with continuity correction or bilateral Fisher exact test (SPSS)

c: Recommendation to relatives [T1-T4] or prescriptions to patients [4]

d: Student t-test (SPSS)

e: Friedman test (SPSS)

f: Cochran test (SPSS)

g: ANOVA test with REGRESS procedure in SUDAAN 7.0 to make use of all data (patients with or without repeated measures)

h: LOGISTIC procedure in SUDAAN 7.0

i: Paired comparisons are calculated only if Global p-value is <0.05. The symbol "≠" means p<0.05. For example, for the first line, "T1≠T2T3≠T4" means that the "Use (% of all categories) of IDB (last period)" is significantly different (p<0.05, after Bonferroni's correction for 6 comparison, i.e., an uncorrected p-value of 0.0085) between 1 week and rest or time-point, but is not different between 1 month and 3 months, and so on

j: Wilcoxon test for paired samples (SPSS)

k: McNemar test (SPSS)

l: t-test with DESCRIPT procedure in SUDAAN 7.0

m: t-test for paired samples (SPSS)

n: chi-square with CROSSTAB procedure in SUDAAN 7.0.

*Not analyzed because the number of data is too low.

### Self-reported frequency of IDB use

The answers related to the question “how many times do you use an IDB” demonstrated considerable heterogeneity (p < 0.001). Of the 20.8% of students who reported using the brushes at T4, 10.0% used them daily. Among IDB users, a significant downward trend in daily use was observed from T1 (32.6%) to T2 (11.5%) and T3 (11.4%) before increasing at T4.

### Knowledge, perceived barriers and facilitators of IDB use

The perceived acceptability, perception of pain and bleeding of the students during the 3 periods are described in Table 2.

Accessibility is perceived as globally satisfactory for all periods. The 66 students who stopped using IDBs between the 3^rd^ month and 12^th^ month did not consider the purchase price of IDBs in the 4^th^ year of study as an essential explanatory model with an average score of 3.05 ± 1.30 on a scale of 5. The discomfort experienced with the use of IDBs is constant across all controlled periods with constant values close to 2, indicating an immediate mean negative perception.

### Perception of interdental brushes

Table 2 shows that the participants' overall impression of "Acceptability, Traumatism and Perceived efficacy" was positive. The acceptability of the students has demonstrated a constant trend according to the study periods, with a level that was globally qualified as average. The same applies to "Traumatism". The perception of efficacy is overall very good, except at 3 months, where 70 students still report using IDBs.

### Link between self-reported application of IDB and recommendation to others

During the 3^rd^ preclinical year, most students would recommend the program to other people (69.8% at T1 and T2), reaching 50% at T3. The average number of individuals reached by students is similar regardless of their membership in non-users group compared to users group except at T2. More importantly, a dramatic increase in the percentage of students either recommending IDBs to relatives or prescribing them to their patients was observed at T4 (Table 2).

The average numbers of individuals who received information from students were 2.83 ± 2.98 (T0 = T1) and 1.60 ± 2.23 (T2, p < 0.01). The rate of recommendation is similar between the users vs. non-users groups, except at 1 month (T1), where the rate is 1.66 times higher for the users group.

At 1 year, the data are significantly different. The percentage of recommendation increased by 200%, with an average number of patients of 11.5 ± 15.3 per student. The focus of the students was on application in clinical practice with homogenization of prescriptions depending on whether they personally used IDBs (p = 0.680).

### Perceived bleeding and clinical bleeding

The interdental brushing reduced interdental bleeding at one week compared with tooth brushing alone at baseline. A reduction of observed bleeding of 48.6% during the first week was observed in the study population (mean change from 40.5 ± 25.1 at T0 (data not shown) vs. 19.7 ± 17, 7 to T1, p <0.001). The effects for the 1-month and 3-month time points do not significantly differ, with a stabilization of the bleeding per student around 20% (Table 3).

Almost all the subjects, at any point in the study, perceived bleeding. The perception of the amount of bleeding increased with time (1.78 (T1) and 2.39 (T4)). This result is correlated with the percentage of the number of sites bleeding (T1 = T2 = T3 vs. T4: 33.9 ± 25.5; p <0.001).

The median students’ scores of the perceived bleeding reduction on a 4-point Likert scale (1 = many times to 4 = never) were statistically significant according to the recall period (Table 3).

**Table 3:** Bleeding in dental students (n = 96).

Variable	1 week [T1]		1 month [T2]		3 months [T3]		1 year [T4]		Global p-value	Paired comparisons^f^
	n	value	n	value	n	value	n	value		
**Perceived bleeding, valid total n (% Yes)**										
All	96	(96.9)	96	(79.2)	96	(85.4)	89	(94.4)	<0.001^c^	T1T4≠T2T3^g^
IDB-non-users	10	(70.0)	35	(82.9)	26	(100)	70	(95.7)	<0.001^c^	T2≠T3^g^
IDB-users	86	(100)	61	(77.0)	70	(80.0)	19	(89.5)	<0.001^c^	T1≠T2T3^g^
p-value^a^		<0.001		0.679		0.010		0.289		
**Perceived bleeding reduction, mean±sd**										
(Likert scale 1 = many times to 4 = never)										
All	89	1.78±0.89	75	2.23±1.09	75	2.13±0.96	71	2.39±1.09	<0.001^d^	T1≠T2T3T4^h^
IDB-non-users	5	3.40±0.89	25	3.04±1.06	18	2.67±0.77	56	2.61±1.11	0.085^d^	*
IDB-users	84	1.68±0.79	50	1.82±0.85	57	1.96±0.96	15	1.60±0.51	0.116^d^	*
p-value^b^		<0.001		<0.001		0.006		0.001		
**Bleeding (% of sites), mean±sd**										
All	96	19.7±17.7	96	23.4±21.6	96	21.7±24.6	96	33.9±25.5	<0.001^e^	T1T2T3≠T4^i^
IDB-non-users	10	31.9±22.3	35	37.1±25.2	26	39.5±27.7	76	38.0±25.0	0.809^d^	*
IDB-users	86	18.3±16.7	61	15.6±14.5	70	15.1±19.8	20	18.5±21.5	0.580^d^	*
p-value^b^		0.020		<0.001		<0.001		0.002		

a: Chi square with continuity correction or bilateral Fisher exact test (SPSS)

b: Student t-test (SPSS)

c: LOGISTIC procedure in SUDAAN 7.0 to make use of all data (patients with or without repeated measures)

d: ANOVA test with REGRESS procedure in SUDAAN 7.0

e: Friedman test (SPSS)

f: Paired comparisons are calculated only if Global p-value is <0.05. The symbol "≠" means p<0.05. For example, for the first line, "T1T4≠T2T3" means that the "Perceived bleeding % Yes" is significantly different (p<0.05, after Bonferroni's correction for 6 comparison, i.e., an uncorrected p-value of 0.0085) between 1 week and 1 month, compared to 3 months and 1 year

g: chi-square with CROSSTAB procedure in SUDAAN 7.0

h: t-test with DESCRIPT procedure in SUDAAN 7.0

i: t-test for paired samples (SPSS).

*Not analyzed because the number of data is too low.

### Need, perception and change in student interdental bleeding over time

The prevalence of interdental sites with bleeding significantly decreased in one week to over 3 months before increasing significantly after one year. IDB-users and IDB-non-users have significantly different trends, regardless of the period under study. In all cases, the use of IDBs is accompanied by a significantly greater reduction the score acquired in the first week (18.3 ± 16.7). IDB-non-users have a similar bleeding index between periods (p = 0.809), with a bleeding ratio that is approximately 2 times greater than the users group.

Perceived bleeding changes significantly depending on the periods of recall. T2 and T3 require more time for the students to declare a lower perception of bleeding, which is still high (79.2 vs. 85.4; p > 0.05). However, the perception of bleeding increases during the period from T3-T4 to a level similar to that observed on the initial date T1 (94.4 vs. 96.9; p > 0.05). The perception of bleeding is similar between the 2 groups, except at 1 week, which is when 100% of users reported bleeding (p < 0.001).

Perceived bleeding reduction on a Likert scale from 1 to 4 favors IDB-users, with a median score that fluctuates approximately 2.8 vs. 1.7 for the non-users group (p < 0.006). Both groups had a slight decrease in bleeding perception at the end of the study, and there was a significant difference between the user vs. non-user groups (2.61 ± 1.11 vs. 1.60 ± 0.51; p < 0.001).

The "Use of IDBs" is associated to the perception of the effectiveness of IDBs at all periods. Those using IDBs perceived the brushes as more effective in terms of the perception of bleeding reduction (at all periods analyzed, better perception of reduction of bleeding in users of brushes) as well as in terms of the objective reduction in bleeding (reduction of bleeding is higher in users) (Table 4).

**Table 4:** Models^a^ (linear regression) for quantitative variables in dental students (n = 96).

		Factors				
	n	IDB use^b^	Female	Tobacco	Baseline bleeding	Adjusted R^2^
		β±se	β±se	β±se	β±se	
**Acceptability of IDB**						
1 week	86	-0.50±0.36	-0.16±0.19	-0.11±0.23	-0.01±0.00	0.08
1 month^c^	61	-0.67±0.18*	-0.16±0.18	-0.22±0.22	-0.01±0.00*	0.19
3 months^c^	70	-0.86±0.21*	0.00±0.19	-0.26±0.22	0.00±0.00	0.18
1 year^c^	20	-0.64±0.26*	-0.13±0.21	0.05±0.25	-0.01±0.00	0.09
**Traum.(sensib. etc) of IDB**						
1 week	86	-0.71±0.42	0.17±0.22	-0.26±0.27	-0.00±0.00	0.08
1 month^c^	61	-0.89±0.21*	0.18±0.20	-0.41±0.25	-0.01±0.00	0.21
3 months^c^	70	-0.90±0.21*	0.14±0.19	-0.37±0.22	-0.00±0.00	0.22
1 year^c^	20	-0.61±0.27*	-0.16±0.21	-0.06±0.25	0.00±0.00	0.13
**Perceived efficacy of IDB**						
1 week	86	-0.55±0.28*	0.26±0.15	0.00±0.18	0.00±0.00	0.12
1 month^c^	61	-0.37±0.15*	0.29±0.15*	-0.07±0.18	0.00±0.00	0.14
3 months^c^	70	-0.56±0.26*	0.25±0.23	-0.04±0.27	0.00±0.00	0.08
1 year^c^	19	-0.15±0.20	-0.07±0.16	-0.06±0.18	0.00±0.00	0.11
**# people recomm. for IDB**^d^						
1 week	96	1.71±1.02	-0.52±0.62	1.08±0.74	-0.01±0.01	0.10
1 month^c^	96	1.52±0.63*	-0.55±0.61	1.27±0.73	-0.01±0.01	0.13
3 months^c^	96	0.20±0.54	0.68±0.48	0.87±0.56	-0.01±0.01	0.09
1 year^c^	96	1.66±4.06	-4.05±3.24	5.07±3.84	0.12±0.06	0.08
**Perceived bleeding reduction**						
1 week	96	-1.53±0.38*	-0.15±0.18	-0.12±0.21	0.00±0.00	0.28
1 month^c^	96	-1.24±0.23*	-0.06±0.22	-0.54±0.27*	0.00±0.00	0.37
3 months^c^	96	-0.70±0.27*	0.02±0.23	-0.08±0.28	0.00±0.00	0.11
1 year^c^	89	-0.89±0.32*	0.36±0.25	-0.12±0.30	0.00±0.01	0.20
**Bleeding (% of sites)**						
1 week	96	-14.98±5.68*	-2.23±3.45	-3.73±4.09	0.26±0.07*	0.22
1 month^c^	96	-21.12±3.93*	0.02±3.83	-2.64±4.59	0.27±0.07*	0.35
3 months^c^	96	-24.98±5.05*	1.32±4.49	0.82±5.27	0.25±0.09*	0.34
1 year^c^	96	-12.87±5.89*	7.21±4.70	2.15±5.57	0.43±0.09*	0.30

*p<0.05.

a: All potential predictor variables are forced into the models. In no case, the correlation between variables was higher than 0.75, indicating no collinearity effects.

b: IDB use at each time period expressed in the line.

c: Conclusions from these models should be taken with care due to missing values.

d: Recommendation to relatives (T1-T4) or prescriptions to patients (T4).

## Discussion

To the best of our knowledge, this study is the first to report on an objective assessment of prophylaxis interdental biofilm interventions in a dental education setting. It also evaluated the consistency of the module in terms of its effectiveness on knowledge and attitudes relevant to the clinical practice of students as well as the content validity of the information. The major teaching objective of the IIOPEP was to promote the daily use of calibrated interdental brushes to disturb biofilms from adolescents to seniors without periodontal diseases using an assimilated technique. Our goal is also to make students aware that the daily use of IDBs has the same clinical value and logic as the use of a toothbrush.

After a highly satisfactory acceptance, a marked decrease of IDB users was observed among students in the aftermath of the curriculum until the 12^th^ month (89.6% to 20.8%). In parallel, an inverse trend was observed for the percentage of student interdental bleeding sites, with marked differences between IDB users and non-users. Conversely, the percentage of students who recommended or prescribed IDB dramatically increased at the 12^th^ month, with a percentage exceeding 90%.

### Practical and clinical significance of the findings

#### Why is there a steady decrease of IDB use?

The persistence of students in IDB use at 3 months could be estimated as moderate. If the initial acceptance by students was considered satisfactory and the duration as moderate, a specific effort must be undertaken for IDB adherence. The initial acceptance of the procedure by the students clearly indicates that there were two distinct groups at the 1^st^ control (7 days), users (89.6%) and non-users (10.4%), without identifying the factors that optimize the commitment of undergraduate students to interdental oral prophylaxis learning.

After a high acceptance rate (89.6%) at the 7^th^ day, the significant decrease observed over time could be explained by a limited perceived need of IDB use in this young and healthy student population. Young people, even in dental medicine, could be less sensitized to personal health problems and self-prevention. The absence of recalls during the last 9 months of follow-up could have contributed to the interruption of use. In addition, the program may have been primarily perceived solely as an encouragement to prescribe IDB to patients rather than for self-use. The reasons for student non-use primarily suggested a decrease in motivation to use IDB at the 12^th^ month. Furthermore, the accessibility, ability to procure calibrated IDBs during the training and assessment year, is perceived as globally satisfactory. The brushes were made available free of charge from T0 to T3 as part of the module; then, in the next year study's time-course, students must finance the purchase of the brushes and obtain them via the traditional distribution networks (Internet, Pharmacy, or Student association). The variations of distributions of the different likert-scales should be explored to identify potential sub-groups of students encountering specific barriers to use.

#### Recommendation of IDB to relatives or prescription to patients

Conversely, the percentage of students who recommended or prescribed IDB dramatically increased at the 12^th^ month. This increase suggests that this goal of the curriculum was satisfactorily achieved, with no difference according to student self-use. This achievement could be explained by the fact that 4^th^ year students of dental medicine are able to prescribe IDB to patients.

### Strengths and limitations

#### What does the curriculum add?

Prescription of IDBs to adults with a healthy periodontium is generally not integrated into the clinical curriculum of the students. Novel content can introduce a surprising or unusual experience, creating a discrepancy in the mind of the student, which can result in short-term arousal of interest to resolve the discrepancy [13]. Similarly, introducing a new concept that demands daily personal investment by students, while challenging at least a 15-year history of exclusive tooth brushing practices, can be considered an “aggressive measure”. The option chosen in the IIOPEP was to give the students the opportunity to increase the perception of their competence on all behavioral aspects as early as possible in the dental curriculum. Content based on individual clinical practices should be relevant and useful to the student in daily life [20].

The training emphasis on oral health education with interdental cleaning for the oral health care provider, as well as on the system that governs the practice approach that supports the preventive approach in managing chronic oral disease, can play a major role in facilitating improvement [4]. Healthy young adults who are training to be future oral health professionals must perform the interventions that they will recommend to their future patients as part of oral prophylaxis [21]. Introducing this module into the curriculum will have a large clinical effectiveness on preventive strategies because students who have acquired knowledge and clinical practice on this topic will be able to efficiently manage the discrepancies in the interdental biofilm of their future patients.

Another pivotal and original aspect of our approach was the long-term assessment of the curricular effect on the self-behavior and practice of the students, which suggested the need to implement corrective actions.

#### Motivation of the students

Because none of the students had previous experience with IDBs, their response to the training reflected their ability to learn and apply the content. However, student motivation is not an isolated concept [22]. Motivation in the classroom is a function of the following five components: the student, teacher, content, method/process, and environment. Aspects of any of these five components could contribute to and/or hinder motivation [20,23]. For example, it is crucial for the team of teachers to be seen as approachable and willing to engage with students both in class and on a one-on-one basis [24]. In our evaluation, the “teacher” component was not considered.

#### Pedagogy

Our method of teaching, based on the principle of reversed pedagogy, is not considered in this study as an adjustment variable. The format used here provided more opportunities for students to engage in critical thinking, independently facilitate their own learning, and effectively interact with and learn from their peers [25].

Factual knowledge is a prerequisite for effective problem solving. However, ‘in real professional practice, factual knowledge is mostly not a goal itself, but only a single aspect of solving professional problems’. Miller describes four levels of assessment: knows, knows how, shows how (competence), and does (performance) [26]. The use of multiple assessment instruments enhances both validity and reliability of results [27]. The students also perceive more satisfaction and motivation with the use of multiple assessment instruments than with the use of a single instrument [28]. Suitability of the assessment instrument(s) can be determined by relating the objectives or outcomes assessed to the different levels of Miller’s pyramid [29].

Our research in dental education presents extensive training in instructional methods, performance assessment, learning theory and topics in oral prophylaxis education. When clinical-problem solving is the level of competence that is required in our specific curriculum, problem-based learning is used as the main method of teaching. The purpose of our course was demonstrated by the time allocated to each topic in teaching; and the level of thinking and competence/performance encouraged by the course objectives. For example, in our prophylaxis module, the curriculum expects the students to solve or prevent clinical problems related to interdental biofilm disorders, which they meet at first contact level.

This publication serves as a reference for dental schools wishing to implement a similar model in their teaching. One of the major difficulties, except in regard to theory, is to provide students clinical skills that are standardized and reproducible. This curriculum should introduce an innovative method to help maintain clinically healthy adults in good oral health, prevent periodontal disease and reduce the risk of systemic diseases. It answers the classic question in medical education: How to transform cutting-edge technologies scientifically proven into clinical competence and action.

The generalization of the study findings is extended by the recommendation of the implementation of an integrated program in oral prophylaxis including brushing technique skills and the clinical skills of daily using interdental brushes in healthcare delivery. This last point is innovative. Our study shows that this training protocol has the capacity, with certain reservations identified, to impact the behavior and actions of future dentists. Educate the next generation of dentists reasoning not in terms of knowledge but in terms of skills and performance, which will need to be fluent in these transformative technology, is essential in the current context of oral health as a major contributor to the global burden of disease and the focus on individual actions to support their health by the people. They will be well skilled and motivated practitioners to educate their patients for oral prophylaxis, prophylaxis they apply to themselves, and to ensure that all patients are able to perform optimal plaque removal.

At least, through these results and this publication, we hope to inform on the fundamental role of advanced prophylaxis in precision medicine and to integrate in their research opportunities this component likely to disturb the interdental microbiota and significantly reduce virulent pathogens as well as gingival bleeding.

#### Main limitation

Our study lacked a control group. There is minimal scientific evidence on patient behavior, patient communication, patient education and methods to support patients in developing effective oral self-care habits [30]. Students are more likely to be intrinsically motivated if they can develop a sense of competence within their education [31,32]. Little attention has been given to exploring whether students’ own competency assessments can be used to predict their clinical performance [33]. The fourth-year clinical phase of the dental program depends on performance in the third-year clinical courses. Therefore, there is a memory effect of the pre-clinical training with a positive projection on new patients, which is independent of the student’s individual care of his own oral interdental hygiene.

A further limitation derived from the design of this study is, when interpreting regression analysis, is that they refer to relations between variables, but one should be cautious regarding causality conclusions. One point to emphasize is the lack of qualitatively-driven input from the students, based on their experiences in this study. The assessment of teaching for all the subjects of the faculty is carried out by an independent organization with students and teachers for which the results are globalized. The only objective criterion in our possession was a request from 2^nd^ year students to advance this training by one year in order to benefit from it as soon as possible.

### Implications

Our study has implications. We have highlighted a dramatic decrease of IDB use in a well-educated population of dental students. Thus, worse results could be expected in the general population in similar age groups.

#### How to encourage IDB self-use

First, a more adapted temporality of recalls should be considered. Repeated and individualized oral hygiene instructions are important components of preventive programs [34]. Although the cohort of students who had fast enrollment at 1 week had relatively stable behaviors at 1 month and 3 months, long-term persistence in terms of use of interdental prophylaxis remains an issue. Corrective actions should be implemented to optimize the precision, content and delay of recall. The selection of an appropriate recall interval is a multifaceted clinical decision that is difficult, if not impossible, to mechanistically evaluate [35]. Evidence for a specific recall interval for all patients in oral prophylaxis is weak [36]. Regarding oral hygiene, no published randomized controlled trials were found on this subject.

#### Understanding the reasons of non-use by students

The corrective measures of the individual, considering specific management of non-acceptance, should be put in place. Communication should be tailored to the needs of the users through diagnosis and risk profiling [37]. Qualitative studies are also needed to better understand the reasons of non-adherence.

#### Benefit of IDB use

Daily use of IDB can significantly reduce interproximal bleeding with an overall preventive fraction of 46% at one week and 72% at 3 months [11]. There is a need to communicate the critical importance of gingival bleeding as an early sign of disease to the students [38]. In our study, the motivations are clearly related to the awareness of the effectiveness of brushes and the perception of bleeding reduction. Oral hygiene-related self-efficacy influences oral hygiene behavior and has the potential to predict oral hygiene outcomes of patients [39]. Motivation and needs are interrelated because the motivation necessary to change a behavior often results when a need is unmet or not satisfied.

A wider dissemination of the curriculum is also desirable, notably to dentists and pharmacists, to better disseminate the preventive message.

## Conclusions

The newly developed and applied curriculum has a positive effect on the interdental hygiene practices of the dental students using IDBs. After the curriculum, more students recommend IDBs to more people despite the fact that only a small number of students continuously use interproximal devices. Motivation to use IDBs is related to the perception of the effectiveness of brushes and the perception of bleeding reduction. Introducing this module into the dental curriculum for pre-clinical students will have a large clinical effectiveness in preventive strategies because the knowledge, skills and clinical practice will enable students to efficiently manage the interdental biofilms of their future patients. At this stage of our experience, we recommend the use of the module on interdental hygiene practices in the oral health curriculum of dental faculties.

## Supporting information

S1 Fig Interdental spaces, colorimetric probe and interdental brushes. (PDF)

S1 Material Regulation, french health procedure code. (DOCX)

S2 Material Translated core questionnaire session 1, 2, 3, 4. (DOCX)

S3 Material Database. (XLSM)
