# Supplementary material for: Educational outcomes of a new curriculum on interproximal oral prophylaxis for dental students
Source: PLoS One. 2018 Oct 10;13(10):e0204564. doi: 10.1371/journal.pone.0204564 (PMC6179232; doi:10.1371/journal.pone.0204564)

Supplementary Fig. 1 Interdental spaces, colorimetric probe and interdental brushes

A. Pack of 5 cylindrical CPS interdental brushes CURAPROX. B. Colorimetric probe. C. Interdental brush

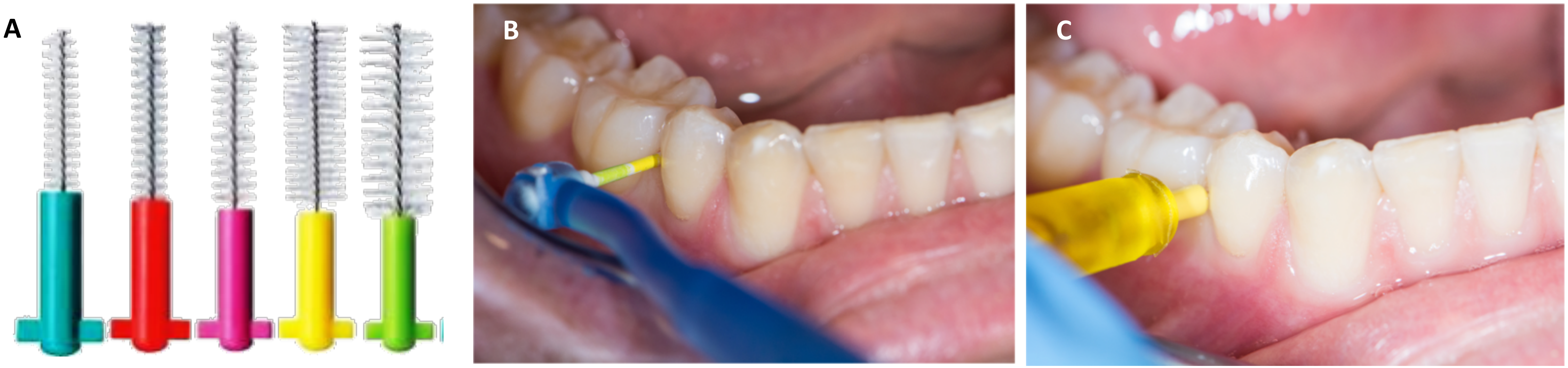

Supplement: S1 Fig — (PDF) [file pone.0204564.s001.pdf]
