# Supplementary material for: Educational outcomes of a new curriculum on interproximal oral prophylaxis for dental students
Source: PLoS One. 2018 Oct 10;13(10):e0204564. doi: 10.1371/journal.pone.0204564 (PMC6179232; doi:10.1371/journal.pone.0204564)
Supplement: S1 Material — (DOCX) [file pone.0204564.s002.docx]

**Supplementary material 1** Regulation, French Health Procedure Code

Medical research studies in France, are classified as non-interventional research in which all the acts are performed and the products used in the usual way, without additional or unusual procedure of diagnosis, treatment or monitoring. Retrospective non-interventional research on existing data with change of purpose and/or existing biological elements is not part of human research (Compliance commitment MR001).

No research involving the human person for the purpose of this title shall be carried out without the organization or practice of healthy or sick persons and to evaluate the practice of health professionals or teaching practices in the field of health. But all the ethical principles and respect for the protection of individuals, the clinical investigations have been conducted according to the principles expressed in the Declaration of Helsinki.

Person in charge of the treatment (promoter) is an independent administrative authority, a legal person who initiates research involving the human person, who manages it, verifies that its funding is provided for and determines the purposes and means of treatment within the meaning of Article 3 of the Data Protection Act.

The scientific research manager is the person designated by the data controller, acting under his responsibility, ensuring the security of the information and its treatment, as well as the respect of the purpose of the latter. He is subject to professional secrecy. In this study it was the coordinating investigator.

The data were analyzed anonymously. The data collection does not allow the identification of the participant. Anonymization process excludes directly nominal data, medico-administrative basic data, identifying genetic data. Nature of data collected authorized relate to an identification number, health, age or date of birth, images, ethnic origin, family situation, level of training, professional life, affiliation system social security (except No.), consumption, tobacco, alcohol, drugs, lifestyle and behaviors, lifestyle, sex life, vital status, quality of life scale... At least, the archiving time of anomy data is 15 years with restricted access.

Implementation and data security implies that the input tool must be secure (user authentication and encryption of data transfer flows). The electronic exchanges of messages must intervene by means of a secure messaging or a dedicated platform with specific rights of access (the simple email is prohibited).
