# Supplementary material for: Educational outcomes of a new curriculum on interproximal oral prophylaxis for dental students
Source: PLoS One. 2018 Oct 10;13(10):e0204564. doi: 10.1371/journal.pone.0204564 (PMC6179232; doi:10.1371/journal.pone.0204564)
Supplement: S2 Material — (DOCX) [file pone.0204564.s003.docx]

**Supplementary material 2** Translated core questionnaire session 1, 2, 3, 4

*This questionnaire was originally administered in french. The translation was made by a native speaker. A back-and-forth translation has been performed in order to decrease translation bias.*

The questions that are asked about changing your behavior since last preclinical session

**Q1.** **Have you used interdental brushes since the last session?** Yes O No O

If "yes", go to question Q4; If "no", go to question Q3 and you will have finished the questionnaire

**Q2.** **For what reasons, did you not use interdental brushes between the two sessions?**

|  | Total agree | Agree | Moderately agree | Disagree | Total disagree | Don’t Know |
| --- | --- | --- | --- | --- | --- | --- |
| Motivation | O | O | O | O | O | O |
| Ability to use IDB | O | O | O | O | O | O |
| Accessibility problem | O | O | O | O | O | O |
| Pain | O | O | O | O | O | O |
| Bleeding | O | O | O | O | O | O |
| Too uncomfortable | O | O | O | O | O | O |
| Cost* | O | O | O | O | O | O |
| Other (specify) : | | | | | | |

**Q3.** **How often did you use interdental brushes?**

| Less than a week **O** | Once a week **O** | 1 day out of 3 **O** | 1 day out of 2 **O** | Once a day **O** | More often **O** |
| --- | --- | --- | --- | --- | --- |

**Q4. Do you have bleeding during the passage of the interdental brushes during the previous session?**

Yes O No O Don’t Know O

If "yes", go to question Q6; If "no" or "do not know", go directly to question Q7

**Q5. Have you seen a decrease in bleeding since the last session?**

| Often O | Occasionally O | Rarely O | Never O | Don’t know O |
| --- | --- | --- | --- | --- |

**Q6. How do you assess the level of use of interdental brushes in terms of … ?**

|  | Very good | Good | Average | Bad | Very bad | Don’t know |
| --- | --- | --- | --- | --- | --- | --- |
| Acceptability | O | O | O | O | O | O |
| Traumatism (sensibility, pain, iatrogenia) | O | O | O | O | O | O |
| Perceived efficacy | O | O | O | O | O | O |

**Q7. Have you recommended IDBs use to relatives or prescriptions to patients?**

Yes O No O

**Q8. If yes, how many people have you recommended IDBs use? ____ _____**

*: Session 4
